# From head to rootlet: comparative transcriptomic analysis of a rhizocephalan barnacle *Peltogaster reticulata* (Crustacea: Rhizocephala)

**DOI:** 10.12688/f1000research.110492.1

**Published:** 2022-05-27

**Authors:** Maksim Nesterenko, Aleksei Miroliubov

**Affiliations:** 1 Department of Invertebrate Zoology, St Petersburg State University, St Petersburg, 199034, Russian Federation; 2 Laboratory of parasitic worms and protists, Zoological Institute of Russian Academy of Sciences, St Petersburg, 199034, Russian Federation

**Keywords:** Rhizocephala, parasitic barnacles, evolutionary transcriptomics, host manipulation, coloniality

## Abstract

**Background**: Rhizocephalan barnacles stand out in the diverse world of metazoan parasites. The body of a rhizocephalan female is modified beyond revealing any recognizable morphological features, consisting of the interna, the system of rootlets, and the externa, a sac-like reproductive body. Moreover, rhizocephalans have an outstanding ability to control their hosts, literally turning them into “zombies”. Despite all these amazing traits, there is no genomic and transcriptomic data about any Rhizocephala.

**Methods**: We collected transcriptomes from four body parts of an adult female rhizocephalan Peltogaster reticulata: externa and main, growing, and thoracic parts of the interna. We used all prepared data for the de novo assembly of the reference transcriptome. Next, a set of encoded proteins was determined, the expression levels of protein-coding genes in different parts of the parasite body were calculated and lists of enriched bioprocesses were identified. We also in silico identified and analyzed sets of potential excretory / secretory proteins. Finally, we applied phylostratigraphy and evolutionary transcriptomics approaches to our data.

**Results**: The assembled reference transcriptome included transcripts of 12,620 protein-coding genes and was the first for both P. reticulata and Rhizocephala. Based on the results obtained, the spatial heterogeneity of protein-coding genes expression in different regions of P. reticulata adult female body was established. The results of both transcriptomic analysis and histological studies indicated the presence of germ-like cells in the lumen of the interna. The potential molecular basis of the interaction between the nervous system of the host and the parasite's interna was also determined. Given the prolonged expression of development-associated genes, we suggest that rhizocephalans “got stuck in the metamorphosis”, even in their reproductive stage.

**Conclusions**: The results of the first comparative transcriptomic analysis for Rhizocephala not only clarified but also expanded the existing ideas about the biology of this amazing parasites.

## Introduction

Rhizocephalan barnacles (Crustacea: Rhizocephala) stand out among metazoan parasites. In the process of adaptation to a parasitic lifestyle, they have changed beyond recognition, losing almost all the structures characteristic of other crustaceans. In particular, they have lost all normal systems of organs, as well asbody axes ^1, 2^. The body of an adult rhizocephalan female is represented by the interna, a system of hollow, ramifying rootlets infiltrating the body cavity of the host (also crustaceans, usually decapods), and the externa, a sac-like body protruding outside the host. The interna is responsible for absorbing nutrients from the host hemolymph and their transportation to the externa ^1^ as well as for interactions with the host ^3, 4^. The externa is a temporary structure thought to be an organ of sexual reproduction ^5^. It usually contains two incorporated dwarf males and a special mantle chamber with developing embryos ^2^. Some rhizocephalans can form several externae, sometimes as many as 2,000 ^6, 7^, which is considered a unique instance of modular/colonial organization among arthropods. Besides their unusual morphology, rhizocephalans have evolved unique life cycle with a characteristic larval stage. The larva injects a few poorly differentiated cells into the host’s hemolymph, and what is left of the larva dies ^8^. Noteworthy, the entire adult body originates from these cells and is thus a newly formed structure ^9^.

In addition to their morphological adaptations and unusual life cycle, rhizocephalan barnacles show a remarkable ability for manipulating the host. These parasites can take control of the molting cycle of the hosts, change their metabolism, behaviour, and even body shape ^2, 10– 20^. Specialized sites responsible for host-parasite interactions have recently been described ^3, 4^, with a network of the host’s neurons enlacing the rootlets of the parasite, but the molecular mechanisms of these interactions remained enigmatic. The authors suggested that the parasite may emit some signal molecules attracting the growth of the host’s neurons ^3^.

A rapid development of high-throughput sequencing technologies has enabled detailed molecular-based biological studies of many living organisms (for example, 21– 25), but until recently, molecular studies on Rhizocephala have been lacking. The research on body heterogony, host-parasite interactions and functional physiology has only been based on morphological and other classical methods.

In an attempt to fill this gap, we made a comparative transcriptomics analysis of different parts of the rhizocephalan female body. Our research object was *Peltogaster reticulata* (Rhizocephala: Peltogasteridae), whose females parasitize hermit *Pagurus minutus* crabs (Crustacea: Decapoda) and form one or, less often, several externae. On the one hand, *P. reticulata* is a typical rhizocephalan, but, on the other hand, it has a lesser degree of modularity than many other representatives of this group ^26^. Thus, it is a particularly convenient research model. In this study, we present the transcriptome-based evidence of molecular and functional heterogeneity of the female rhizocephalan body. We also show that the ovary is diffused throughout the interna, and host’s motor neuron axon are attracted to the interna rootlets. Phylostratigraphy and evolutionary transcriptomic analysis were performed for Rhizocephala for the first time. Our results make it possible to trace evolutionary trends in the *P. reticulata* gene set.

## Methods

### Sampling

Hermit crabs *Pagurus minutus* Hess, 1865 infected with *Peltogaster reticulata* Shiino, 1943 were collected in the Sea of Japan (Marine Biological Station “Vostok” of the Institute of Marine Biology of the Russian Academy of Sciences) (N: 42.893720, E: 132.732755). All the parasites were adults with fully developed externae.

**Figure 1.**
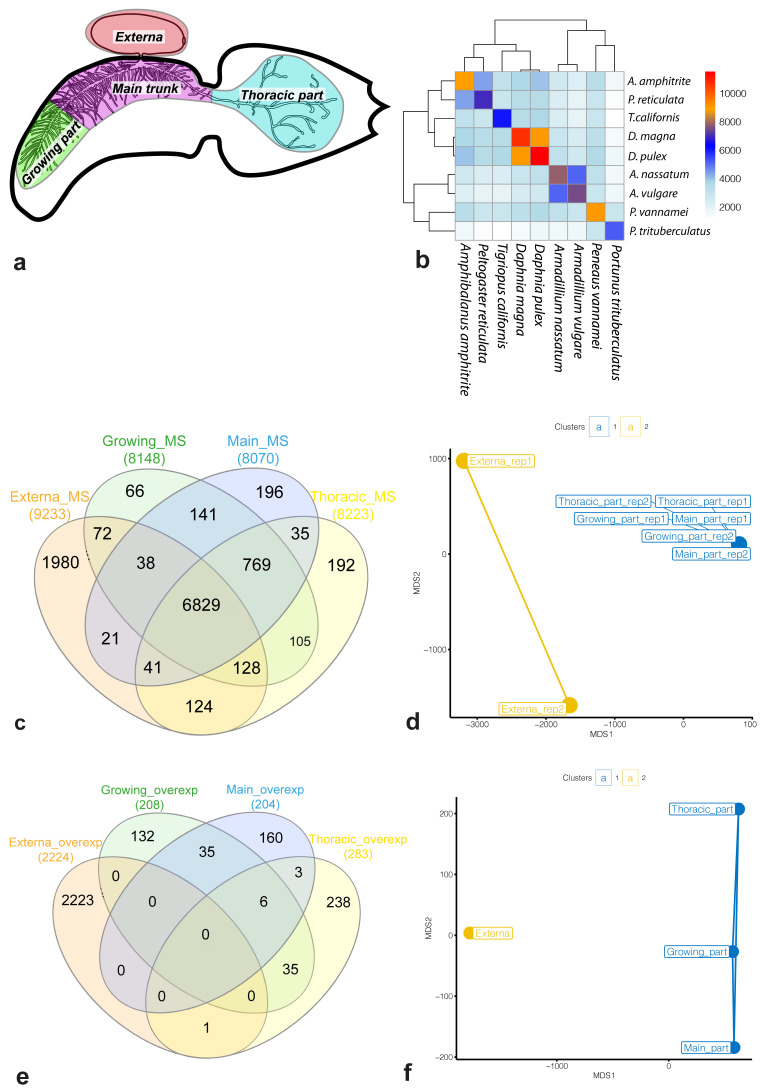
Molecular signatures of female *Peltogaster reticulata*. ( **a**) Generalized scheme of female *P. reticulata* in the host. Colour sectors indicate the body parts examined in our study: externa (red), growing part of interna (green), main trunk of interna (purple), and thoracic part (blue). ( **b**) The number of common OMA groups. The colour key on the heatmap shows the number of shared OMA groups between species. ( **c**, **e**) Venn diagram for a set of genes either included in molecular signatures of the body parts ( **c**) or over-expressed in the body parts ( **e**). ( **d**, **f**) Multidimensional scaling (MDS) plots for molecular signatures ( **d**) and sets of over-expressed genes ( **f**). Different clusters in MDS plots are marked with colours. Abbreviations: MS/overexp – the molecular signature or set of over-expressed genes for the body part, respectively; rep1/2 – biological replication identifier.

The infected crabs were dissected in filtered sea water. The parasite was removed from the host’s body cavity and the remains of the host tissues were isolated from the interna. The body of each parasite was divided into four parts: 1) the externa, 2) distal part of the main trunk (the growing part), 3) the main part of the interna trunk (the main trunk), 4) the part of interna from the thorax of the host (the thoracic part) ( Figure 1). For each body part the pooled sample was prepared, containing material from five parasitic individuals in two biological replicates. The samples were collected into the centrifuge tubes and frozen at -80 °C in IntactRNA (Evrogen, Moscow, Russia) reagent according to the manufacturer`s protocol.

Before RNA isolation, the IntactRNA-fixed samples were rinsed in 0.1M phosphate-buffered saline (PBS). The total RNA was isolated using Quick-RNA MiniPrep (R1054, Zymo Research, Irvine, California, USA) according to the manufacturer protocol. The libraries were synthesized using NEBNext Ultra Directional RNA Library Prep Kit for Illumina (E7760, New England BioLabs, Ipswich, Massachusetts, USA) according to the manufacturer protocol. Paired-end sequencing was carried out using Illumina HiSeq 2500 instrument (Illumina, San-Diego, California, USA).

Sampling was conducted in accordance with the European Community Council Directive of November 24, 1986 (86/609/EEC). All possible effort was made to minimize the number of animals used.

### Preparation of reads libraries and de novo transcriptome assembly

The primary quality control of paired-end reads libraries was manually assessed using FastQC (v0.11.5) [ https://www.bioinformatics.babraham.ac.uk/projects/fastqc/]. The potential sequencing error identification and correction was performed by Karect ^27^ (v1.0) [ https://github.com/aminallam/karect] with the following parameters: --celltype=diploid –matchtype=hamming. Trimmomatic (v0.39) [ http://www.usadellab.org/cms/?page=trimmomatic] was used for removing the sequencing adaptors (ILLUMINACLIP:$ADAPTERS:2:30:10:2:TRUE), low-quality read regions (SLIDINGWINDOW:4:20 MAXINFO:50:0.8) as well as reads with a length less than 25 nucleotides (MINLEN:25). Since libraries preparation and sequencing were performed in the laboratory where researchers also work with human medical samples, we checked the parasite data for the presence of the read pairs with a high identity to the *Homo sapiens* reference transcriptome (GENCODE v.31) using BBTools (v37.02).

The prepared libraries were pooled and used for *de novo* reference transcriptome assembly using Trinity ^28^ (v2.5.1) with k-mer size and required minimal contig length equal to 25 and 200 nucleotides, respectively. The assembled contigs were renamed by adding the four-digit tag of the species, “Pret”, at the beginning of IDs. Isoforms were clustered on all assembled contigs using CDHIT-est ^29^ (v4.7) [ http://weizhong-lab.ucsd.edu/cd-hit/] and a sequence identity threshold equal to 95% (-c 0.95), accurate mode (-g 1), and both +/+ and +/- strands alignments (-r 1). TransRate ^30^ (v1.0.1) was used for the quality assessment of clustered sequences. Only the contigs classified as “good” by TransRate ^30^ were included in further analysis.

### Removal of potential contamination

The 18S and 28S ribosomal RNA sequences were searched using RNAmmer ^31^ (v1.2) from the Trinotate pipeline (v3.1.1). The sequences obtained this way were compared with the NCBI nucleotide database with BLASTn ^32^ (v2.6.0+) to identify their possible sources.

We used Model-based Categorical Sequence Clustering (MCSC) Decontamination method ^33^ for removing potential contamination, with “Arthropoda” as the target taxon and the clustering level equal to 5 (32 clusters). The parsing of BAM-files with reads alignment results created by Bowtie2 ^34^ was performed to extract only reads pairs that mapped to the decontaminated set of contigs.

### Quantification of gene expression levels and identification of encoded amino acid sequences

Salmon ^35^ (v1.0.1) was used for expression level quantification (-l ISF –discardOrphans –seqBias –gcBias –validateMappings). The expression quantification results and the transcripts-to-genes map from the Trinity output were provided to the “tximport” package for R to obtain expression levels of genes. The tables with both unaveraged transcripts-per-million (TPM) values and TPM values averaged between biological replicates were prepared. Only the sequences with expression levels ≥ 1 TPM in at least one sample were included in further analysis.

TransDecoder (v5.5.0) was used for the identification of the amino acid sequences encoded by assembled contigs. Firstly, the long open reading frames with a length ≥ 100 amino acids (aa) and products of its translation were found. Secondly, identified proteins were compared with the NCBI non-redundant ( DIAMOND BLASTp ^36^ (v0.9.22.123), e-value = 1e-3) and the PfamA ^37^ ( HMMscan (v3.1b2)) databases. Thirdly, the comparison results were provided to the TransDecoder to identify the likely coding regions and to obtain the probable set of proteins.

### Reference gene set preparation

In our analysis, we focused only on genes that successfully passed two filters: 1) noticeable expression level (i.e., the expression is ≥ 1 TPM in at least one sample) and 2) encoding of the proteins with a length greater than or equal to 100 amino acids. Only the longest protein and its coding transcript were selected as representatives for each gene and referred to as “reference sets”. The completeness of the protein reference set was evaluated by comparison with the database of single-copy orthologues of Metazoa (odb-9) using BUSCO ^38, 39^ (v3.0.1) (e-values = 1e-3, mode = proteins).

### Sequence annotation

For the annotation of the genes, their nucleotide and amino acid sequences were compared with publicly available databases: NCBI nucleotide collection (nt), NCBI non-redundant (nr), and SwissProt ^40^. The similarity search was carried out with BLASTn megablast ^32^ (nt) and the sensitive mode of DIAMOND BLASTp ^36^ (amino acid databases), with an expected value (e-value) threshold equal to 1e-3 and a limit up to 10000 for the number of description and alignments ^41^. The best BLAST hits (BBH) were selected with a custom script.

The potential domain architecture of the proteins was identified using the PfamA database (HMMScan) and custom script. The proteins were also analysed using the eggNOG-mapper web-resource ^42^ (v2) [ http://eggnog-mapper.embl.de] with default parameters.

### Identification of orthogroups

Orthogroups were identified with the use of OMA standalone program (v2.5.0) ^43^ in three steps. Firstly, the reference proteomes of *Amphibalanus amphitrite* Darwin, 1854 (UP000440578), *Armadillidium nasatum* Budde-Lund, 1885 (UP000326759), *Armadillidium vulgare* Latreille, 1804 (UP000288706), *Daphnia magna* Straus, 1820 (Strain: Xinb3) (UP000076858), *Daphnia pulex* Leydig, 1860 (UP000000305), *Penaeus vannamei* Boone, 1931 (UP000283509), *Portunus trituberculatus* Miers, 1876 (UP000324222), and *Tigriopus californicus* Baker, 1912 (UP000318571) were downloaded from the UniProt ^40^ database [accessed 21 October 2021]. Only the sequences with a length equal to or more than 100 amino acids were analysed. The OMA standalone was run with default parameters with the “bottom-up” algorithm for inference of hierarchical orthologous groups (HOGs), without a phylogenetic tree, but with the identification of two *Daphnia* species as an out-group. Secondly, we reconstructed the phylogenetic tree following the protocol by Dylus *et al.* ^44^. Briefly, using the filter_groups.py provided, we selected OMA groups that included at least eight of the nine crustacean species involved in the analysis. Then, using MAFFT ^45^ (v7.487), multiple protein alignment in each orthogroup was performed (--maxiterate 1000 -localpair). The alignments were concatenated into a supermatrix using the concat_alignments.py. The selection of suitable sites in the supermatrix was carried out using tramAl ^46^ (-automated1). We used the ProtTest program ^47, 48^ (v3.4.2) to determine the most appropriate sequence evolution model. The phylogenetic tree was reconstructed using the IQ-TREE ^49, 50^ (v2.1.4-beta) with the following parameters: -m LG+I+G+F --seed 12345 -B 1000 --nmax 1000. The consensus tree was rooted by the out-group using the “ ape” ^51^ package for R. Thirdly, the phylogenetic tree was used when the OMA standalone ^43^ was re-run with default settings. The construction of a heatmap with the number of common OMA groups between the studied species was performed in RStudio using the “ggplot2” (v3.3.5), “pheatmap” (v1.0.12), and “RColorBrewer” (v1.1-2) libraries.

### Reference gene set expression analysis

We defined the “molecular signature” of a body part as a set of genes with an expression level ≥ 2 TPM in the body part. The expression threshold value was chosen in accordance with the results of studies by Wagner, Kin, and Lynch, according to which “genes with more than two transcripts per million transcripts (TPM) are highly likely from actively transcribed genes” ^52^. If a gene had an expression ≥ 2 TPM in all the body parts, we classified it as “commonly expressed”.

Significant variation of gene expression between samples was detected using the “ RNentropy” library ^53^ (v1.2.2) for R. The analysis was carried out using a table with unaveraged TPM values between replicates. We used the corrected global sample specificity test P < 0.01 according to the Benjamini-Hochberg method, and local sample specificity test P < 0.01.

The overlaps between the molecular signatures and the sets of over-expressed genes were visualized with the InteractiVenn ^54^.

### Multidimensional scaling

We performed a multidimensional scaling (MDS) analysis for the molecular signatures and the sets of over-expressed genes. The presence / absence matrices were used as input. It was indicated in the matrix for each gene (row) whether the gene was included in the molecular signature of the body part or had an increased expression in it (“1”) or not (“0”). The optimal number of clusters was determined using the “silhouette” method implemented in the “ factoextra” library (v1.0.7) for R. We used the metaMDS function from the “ vegan” library (v2.5-7) with the following parameters: distance = "manhattan", try = 100, trymax = 100000, autotransform = FALSE, binary = TRUE, k = the optimal number of clusters. The seed was set to 1234 both when the optimal number of clusters was determined and in MDS. To visualize the results, we used the ggscatter function from “ ggpubr” (v0.4.0) library for R.

### Potential excretory/secretory proteins (ESP) identification and analysis

The *in silico* identification of potential ESP was performed according to the pipelines described by Garg and Ranganathan ^55^. Firstly, all proteins from the reference set were analysed with SignalP ^56^ (v5.0b). Based on the analysis results, the proteins were divided into potential “classical” (SP ≥ 0.5) and “non-classical” (SP < 0.5) ESP. Secondly, the “non-classical” ESP were analysed using SecretomeP ^57^ (v1.0) [ https://services.healthtech.dtu.dk/service.php?SecretomeP-2.0]. Only proteins that had NN-scores ≥ 0.9 and were simultaneously predicted not to contain a signal peptide were selected. Thirdly, all potential ESP were scanned for the presence of the mitochondrial transit peptide with TargetP ^58^ (v2.0). The proteins with this signal were excluded. Fourthly, transmembrane hidden Markov model (TMHMM) ^59^ (v2.0c) [ https://services.healthtech.dtu.dk/service.php?TMHMM-2.0] was used to model and predict the location and orientation of transmembrane domains in proteins, and only proteins without them were considered as potential ESP. Out of these potential ESP, we selected only those that were included in at least one molecular signature. The overlap analysis between ESP sets were performed using InteractiVenn ^54^.

We ran all against all BLASTp searching using DIAMOND ^36^ (--evalue 1e-3) for both classical and non-classical ESP. Similarity search results were used in Silix ^60^ (v1.2.11) (-r 0.9), which assigns proteins to putative gene families. For annotation, all ESP were compared against the NeuroPep ^61^ and MetazSecKB ^62^ databases using DIAMOND BLASTp ^36^ with the following parameters: --sensitive --max-target-seqs 10000 –evalue 1e-3. The best BLAST hits were selected using a custom script.

### Gene set enrichment analysis (GSEA)

The GSEA using “ topGO” library (v2.40.0) for R was performed for 1) whole molecular signatures, 2) sets of over-expressed genes, and 3) sets of potential “classical” and “non-classical” ESP. Only the Gene Ontology (GO) terms describing biological processes were considered. We used Fisher’s exact test and extracted only the terms including at least 10 significant genes (GO terms with p-value <0.01) from the results. Redundancy was reduced with the “ rrvgo” library (v1.0.2) . We used the minus log10-transformed p-values as scores, org.Dm.eg.db as database, relevance as similarity measures methods, and 0.7 as the threshold for reduceSimMatrix. We used the “ wordcloud” (v2.6) library for R to build word clouds based on the redundancy reduction results. The more often the parental bioprocess was found in the list of enriched bioprocesses, the larger the word size. Each bioprocess was assigned a colour from the “viridis” (v0.6.1) palette package.

### Phylostratigraphy and Transcriptome Age Index (TAI) measuring

The phylostratigraphy analysis of the *P. reticulata* reference protein set was performed using the “ phylostratr” package ^63^ (v0.2.1) for R. We used reference proteomes of Crustacea prepared beforehand as well as the prebuilt dataset of prokaryotes, human, and yeast. Similarity search between proteins was carried out with BLASTp ^32^ (v2.6.0+). The tables with BLAST results in “6” output format were used as input for “phylostratr” for protein distribution between phylostrata: 1) “Cellular organisms”, 2) “Eukaryota”, 3) “Opisthokonta”, 4) “Metazoa”, 5) “Eumetazoa”, 6) “Bilateria”, 7) “Protostomia”, 8) “Ecdysozoa”, 9) “Panarthropoda”, 10) “Arthropoda”, 11) “Mandibulata”, 12) “Pancrustacea”, 13) “Crustacea”, 14) “Multicrustacea”, 15) “Hexanauplia”, 16) “Cirripedia”, and 17) “ *Peltogaster reticulata*”.

The phylostratigraphic composition was analysed for *P. reticulata* reference gene set, the set of genes with noticeable (≥ 2 TPM) expression in all the female body parts considered, the sets of overexpressed genes as well as the sets of genes encoding potential “classical” and “non-classical” ESP. The results were visualized using “ggplot2”, “viridis” (v0.6.1), and “reshape” (v0.8.8) libraries for R.

Transcriptome Age Index (TAI) definition was performed for *P. reticulata* body parts using phylostratigraphy results and tables with averaged TPM-values. The analysis was carried out using the “ myTAI” ^64^ (v0.9.3) package for R. Genes with an expression level < 2 TPM at all the body parts were excluded. The analysis was carried out on log2(TPM + 1) transformed values. We used the FlatLineTest function to quantify the statistical significance of the global TAI pattern. For analysis with the use PlotRE and PlotBarRE functions, the phylostrata were divided into two groups: “before” (phylostrata 1-13), and “after” (phylostrata 14-17) the division of Crustacea.

Using the pMatrix function from “myTAI” ^64^, the contributions of genes to the TAI of body parts were determined. For each body part, 500 genes with the largest contribution were selected out of the genes with the GO annotation. Further, GSEA for the selected gene sets was performed similarly to GSEA for the molecular signature.

### Histology

For histological and light-microscopic examination, the dissected internae were fixed with Bouin solution (picric acid (trinitrophenol) 71.5%, paraformaldehyde 24% and acetic acid 4.5%). Paraffin sections 5 μm thick were made using standard histological methods with the help of a Leica RM-2265 microtome and stained with hematoxylin-eosin. The sections were examined under a Leica DM2500 microscope. The photos were taken with a Nikon DS-Fi1 camera and processed with ImageJ software (FiJi ^65^).

### Confocal laser scanning microscopy (CLSM)

Samples of interna for immune labelling were fixed with 4% paraformaldehyde (PFA; Sigma-Aldrich) in PBS (Fluka) at 4 °C for four hours, and then rinsed three times with PBS. Before the immunocytochemical staining, the fixed material was incubated with PBST (PBS + 0.1 % Triton-X100; Sigma-Aldrich) during 24 hours at 4 °C. Then, the samples were incubated in primary antibodies and anti-acetylated α-tubulin (Sigma Aldrich, Germany, T6793, produced in mice) and anti-serotonin (Sigma Aldrich, Germany, S5545, produced in rabbit) for three days. After incubation the specimens were rinsed in PBS three times and incubated in secondary antibodies anti-mouse IgG CFTM 633 (Sigma Aldrich, Germany, SAB4600138) and anti-rabbit IgG CFTM 488A (Sigma Aldrich, Germany, SAB4600030).

The specimens were rinsed with PBS three times and stained with the DAPI nuclei stain (1 μg/ml; Sigma-Aldrich) for 30 min, rinsed in PBS and mounted in DABCO-glycerol. The samples were examined using the confocal laser scanning microscope Leica TCS SP5 in the Resource Center “Microscopy and Microanalysis” of the Research Park of Saint Petersburg State University. The images were processed by ImageJ software (FiJi ^65^).

### Scanning electron microscope (SEM)

Specimens for SEM were fixed at 4 °C in 2.5% glutaraldehyde, dehydrated in ethanol series and acetone, critical point-dried in a Hitachi critical point dryer HCP- 2, mounted on stubs, coated with platinum with the use of a Giko IB-5 Ion coater, and viewed under a FEI Quanta 250 scanning electron microscope in the “Taxon” Research Resource Center of the Zoological Institute of the Russian Academy of Sciences.

## Results

### The *de novo* assembled transcriptome was characterized by high quality and completeness of assembly

The transcriptomes of the whole body ( Figure 1a), the thoracic part of the interna ( Figure 1a), the growing part of the interna ( Figure 1a) and the main trunk of the interna ( Figure 1a) as well as that of the externa ( Figure 1a) of adult *P. reticulata* were collected and sequenced in two biological replicates. More than 87% of read pairs remained in all the paired-end libraries after the removal of adapters, poor-quality regions, and short sequences (Table S1, *Underlying data* ^66^). The potential contamination with human-derived sequences did not exceed 4.1% from the total number of trimmed read pairs in each library (Table S1, *Underlying data)*.

All prepared libraries were merged, and the resulted libraries were used as input for Trinity software. In general, 353,130 contigs with lengths greater or equal to 200 nucleotides were assembled *de novo*. After the clusterization of similar sequences, the *P. reticulata* transcriptome included 267,188 contigs. TransRate assembly and optimal scores made up 0.3331 and 0.3835, respectively. More than 95% (256047/267188) of the contigs were well-assembled (“good”) according to the TransRate quality control results. The completeness analysis for “good” contigs using a database of the metazoan single-copy orthologues revealed that 96.3% (Single: 57.8%, duplicated: 38.5%) of the orthologues were assembled completely.

### *Peltogaster reticulata* reference transcriptome included transcripts of 12,620 protein-encoding genes

Given the parasitic lifestyle of *P. reticulata*, the assembled transcriptome was checked for the presence of potential contamination. According to the RNAmmer analysis results, eight and nine sequences could be classified as 18S and 28S ribosomal RNAs, respectively. The comparison with the NCBI nucleotide database revealed that the contigs successfully aligned with ribosomal sequences from Alveolata (HQ891115.2), Fungi (AY382649.1; CP033152.1; CP030254.1; GQ336996.1; MF611880.1), and Metazoa (AY265359.1; EU082415.1; KY454201.1; EU370441.1; KU052603.1). Among the metazoan hits, only sequences from Crustacea were identified. The identity percentage with the database-derived 18S host (KY454201.1) and parasite (EU082415.1) sequences made up 88% (990/1,123) and 99% (1,200 / 1,201), respectively. In this study, we used the MCSC hierarchical clustering algorithm for removing potential contamination from the assembled transcriptome. The decontaminated transcriptome included 80,779 contigs and contained 81.6% (Single: 56.9%; duplicated: 24.7%) of completely assembled single-copy metazoan orthologues. The number of paired-end reads successfully aligned to selected contigs varied from 10.74 (externa, first replicate) to 30.64 million (the thoracic part of the interna, second replicate) (Table S1, *Underlying data)*

The sequence expression level quantification was performed with Salmon by mapping selected read pairs to the decontaminated transcriptome. The mapping rate ranged from 85% to 91%. The transcript-to-gene map was used to obtain gene expression levels in TPM values. After excluding genes with a low activity in all analysed samples (expression level < 1 TPM), the dataset contained TPM values for 20,980 contigs. According to the TransDecoder results, 32,990 contigs encoded proteins with lengths ≥ 100 amino acids.

Only the protein-coding genes with a noticeable expression were involved in further analysis. After filtering by expression level (≥ 1 TPM in at least one sample) and encoded protein lengths (≥ 100 aa), we obtained the reference gene set for *P. reticulata* containing 12,620 sequences. For each gene, the longest protein encoded by its splice variants was selected as a representative sequence. The comparison with a single-copy metazoan orthologues database revealed that in the reference protein set of *P. reticulata* 75.6% of the orthologues were assembled completely, whereas 21% of the sequences were absent.

### Most of the sequences from the reference sets were annotated successfully

The prepared reference sets were compared with publicly available databases. According to the results, 3,399, 8,502, and 9,690 genes had hits with NCBI nucleotide, SwissProt, and NCBI non-redundant databases, respectively. The overlap analysis revealed that 3,240 genes were successfully annotated using each database. Moreover, 6,090 genes belonged to at least one GO terms. The domain architecture of the encoded protein was identified for 8,803 genes based on the comparison with the PfamA database. Annotation results are presented in Table S2 ( *Underlying data* ^67^).

### The proteins set of *P. reticulata* was similar to the reference proteome of another cirripede barnacle, *Amphibalanus amphitrite.*

The identification of orthogroups in *P. reticulata* and other Crustacea involved in this study was carried out in three stages. First, the proteomes of *P. reticulata* and eight reference crustacean species were analysed using OMA standalone. As a result, 24,840 OMA groups were discovered.

Secondly, a phylogenetic tree of the studied crustacean species was constructed based on the results (Figure S1, *Extended data* ^68^). We selected 609 OMA groups, containing at least eight out of nine species. The multiple protein alignment results in each of the OMA groups were concatenated into a supermatrix. After site selection, the final supermatrix contained 282,907 sites. In the resulting phylogenetic tree, *P. reticulata* was united into the same taxon with another cirripede barnacle, *Amphibalanus amphitrite*, with full support (Figure S1, *Extended data* ^68^).

Thirdly, the resulting tree was used to refine the search results for orthogroups. More than 20,000 orthogroups were found: 24,840 and 20874 OMA and HOGs were identified, respectively. Figure 1b shows the number of common OMA groups for pairs of species. *P. reticulata* had the largest number of "common" OMA Groups (4,354) with *A. amphitrite*. For comparison, *P. reticulata* had no more than 3,490 “common” OMA groups with other crustacean species, the smallest overlap being found with *Portunus trituberculatus* (1,262 OMA groups).

### The externa was clustered separately from the interna based on the gene expression analysis results

We defined the molecular signature of a body part as a set of genes with an expression ≥ 2 TPM in the body part considered. Each molecular signature included at least 8,000 genes: the main trunk of interna (8,070 genes), the growing part (8,148), the thoracic part (8,223), and externa (9,233) (Table S3, *Underlying data*). Approximately 54% (6,829 / 12,620) of the genes from the reference set were included into molecular signatures of all body parts ( Figure 1c). Figure 1c shows significant overlaps between the parts of the interna and an almost 10-fold difference in the number of “specific” genes between the interna parts and the externa. Based on the gene expression, the body parts were divided into two clusters ( Figure 1d). The first cluster contained all parts of the interna, while the second cluster contained only two replicates of the externa.

According to the differential expression analysis results, the number of over-expressed genes varied from 204 (the main trunk of the interna) to 2,224 (the externa) (Table S3, *Underlying data*). The number of genes with an increased expression in the interna did not exceed 283. Figure 1e shows that (i) only 1 gene was over-expressed both in the externa and in the interna, (ii) the number of “shared” over-expressed genes between different interna parts ≤ 35, (iii) only six genes were over-expressed in the whole interna. The externa clustered separately from the interna, and all interna parts were remote from each other ( Figure 1f).

The results of identification of molecular signatures and differentially expressed genes are presented in Table S3 ( *Underlying data* ^69^).

### GSEA results and histological studies indicate the presence of germ-like cells in the interna

**Figure 2.**
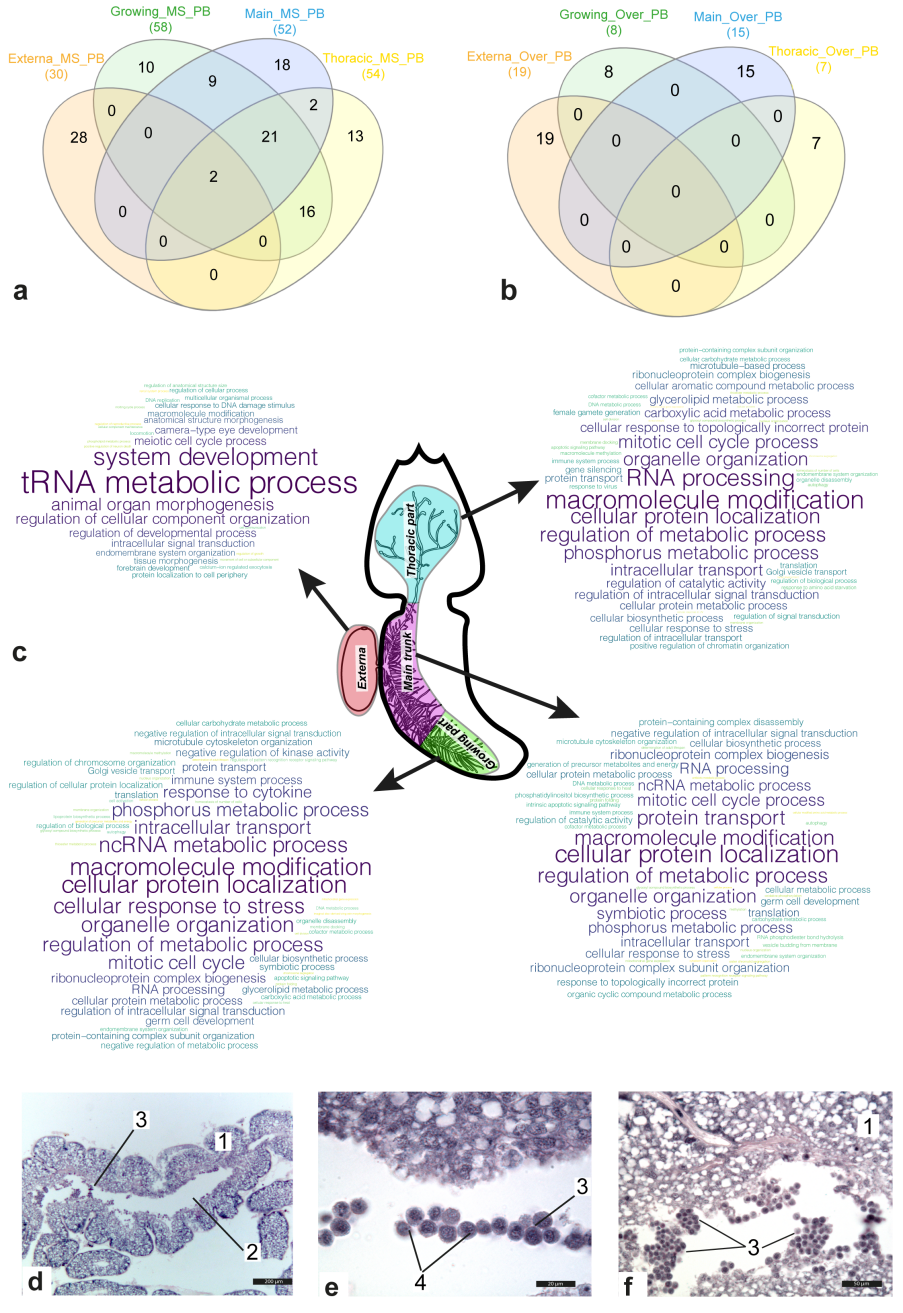
Gene set enrichment analysis (GSEA) results for the gene sets. ( **a**, **b**) Venn diagram for sets of parental bioprocesses enriched with either genes included in the molecular signature of the body part ( **a**) or genes over-expressed in body part considered ( **b**). ( **c**) Clouds of enriched parental bioprocesses for the body parts. Most often, the bioprocess was found in the list of enriched bioprocesses, the larger the word size. Abbreviations: MS/Over – bioprocesses enriched with genes included either in molecular signature or in sets of over-expressed genes; PB – parental bioprocesses. ( **d**– **f**) Histological sections of the main trunk of *P. reticulata* 1 - the wall of the main trunk; 2 - central lumen; 3 - groups of the floating cells; 4 - Nuage body. Scale bars: d - 200µm, e - 20µm, f - 50µm.

Figure 2a and b show Venn diagrams for lists of bioprocesses enriched with genes included in the molecular signatures ( Figure 2a) and genes with increased expression ( Figure 2b) in the female body parts considered. In contrast to the externa, where many active bioprocesses were associated with development, bioprocesses enriched in the interna were mainly connected with metabolism. Comparative analysis results revealed that 50 bioprocesses were active in at least two parts of the female body. Among them were “mitotic cell cycle process” (main trunk and the thoracic part of interna), “cell division” (growing and thoracic part of interna), “homeostasis of number of cells” (same), “apoptotic signalling pathway” (same), “symbiotic process” (growing and main trunk of interna), “determination of adult lifespan” (same), “immune system process” (the growing part, the main trunk, and thoracic part of interna), and “autophagy” (same).

The “germ cell development” bioprocess was enriched in the main trunk and the growing part of the interna, whereas “female gamete generation” was only found among the lists of enriched bioprocesses in the thoracic interna part ( Figure 2c). At the same time, according to our results, meiosis probably only occurred in the externa ( Figure 2c). The results of histological studies also confirmed the presence of germ-like cells in the central lumen of the rootlets of the interna. Groups of floating small round cells with a high nuclear cytoplasmic ratio were found in the central lumen of the main trunk and peripheral rootlets ( Figure 2d–f). A Nuage body ( Figure 2d–f), which is a marker of germ cells, was present in each cell next to the nucleus.

No common bioprocess was found in different parts of the female body, enriched with over-expressed genes. The genes with over-expression in the externa were involved in bioprocesses associated with cuticle transformation and development of the nervous system. In the growing interna part, over-expressed genes were involved in “gland development,” “response to nutrient levels”, “organic acid transport”, as well as lipid and fatty acid metabolic processes. In addition to various metabolic processes, “determination of adult lifespan” and “intrinsic apoptotic signaling pathway” were also found among a set of enriched bioprocesses for the main trunk. Bioprocesses associated with responses to various stimulus (bacterium/oxygen-containing compound/wounding), as well as “interspecies interaction between organisms”, “ion transmembrane transport”, “cellular homeostasis”, and “aging” were classified as “enriched” in the thoracic part of the interna.

All GSEA results are presented in Table S4 ( *Extended data* ^70^).

### Hundreds of genes encoding potential excretory/secretory proteins (ESP) were identified

**Figure 3.**
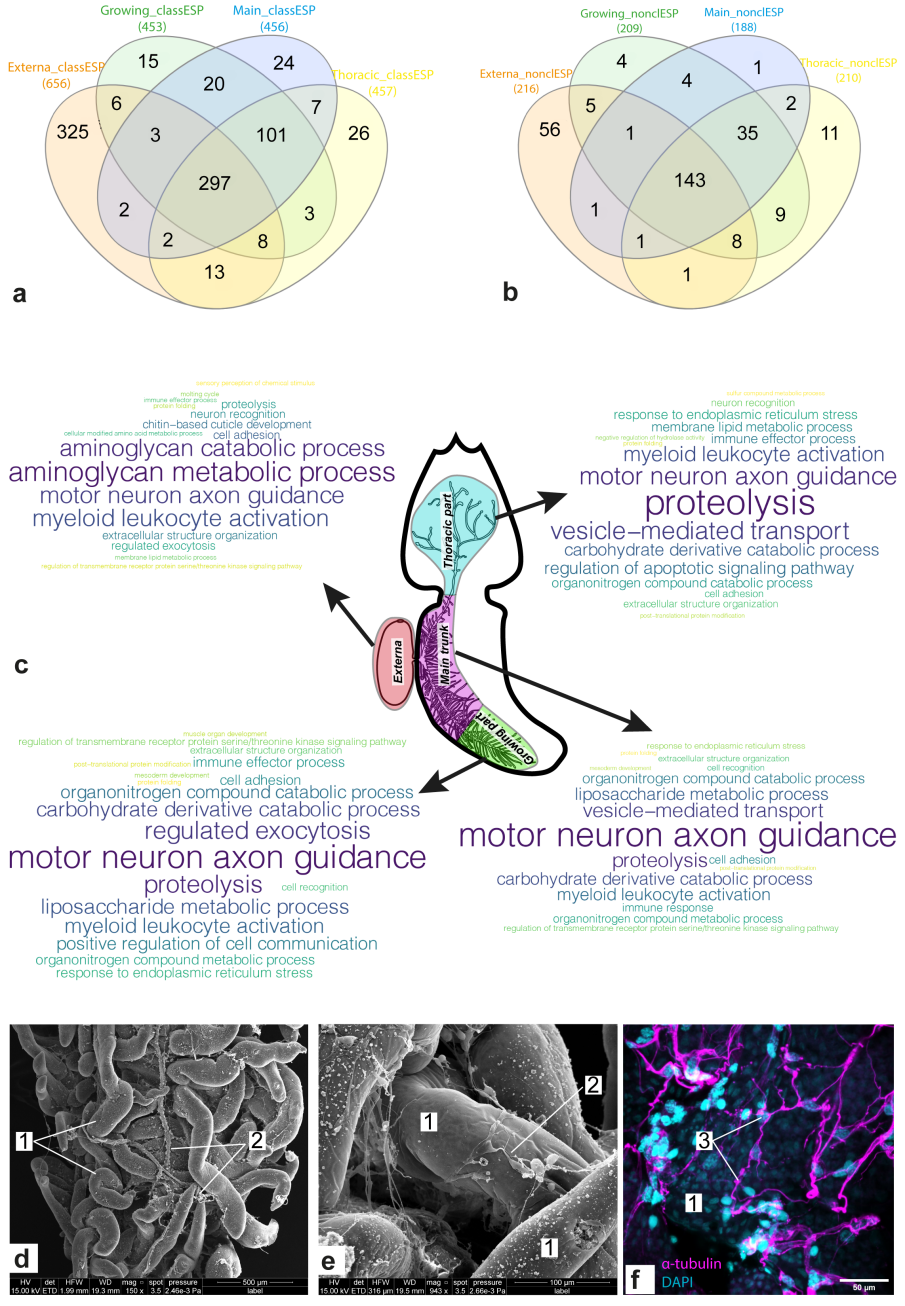
Gene set enrichment analysis (GSEA) results for the identified sets of potential excretory/secretory proteins (ESP). ( **a**, **b**) Venn diagram for sets of potential “classical” ( **a**) and “non-classical” ( **b**) ESP encoded by genes from molecular signatures of the body parts. ( **c**) Clouds of parental bioprocesses enriched by genes encoding “classical” ESP. Most often the bioprocess was found in the list of enriched bioprocesses, the larger the word size. ( **d**– **e**) Scanning electron microscope (SEM) photos of the interna of *P. reticulata*, 1 - rootlets; 2 – host tissues enlacing rootlets. ( **f**) Confocal laser scanning microscopy (CLSM) photo of the interna of *P. reticulata*, scale bar 100µm, 3 - host tissues stained with antibodies against α-tubulin. Abbreviations: class/nonclassES – “classical” and “non-classical” ESP, respectively.

The identification of potential ESP was performed *in silico*. We found 852 "classical" and 282 "non-classical" ESP, which, respectively, had or did not have classical N-terminal signal peptides. Figure 3a, b shows Venn diagrams for the sets of genes encoding “classical” ( Figure 3a) and “non-classical” ( Figure 3b) ESP, respectively, which were included in the molecular signatures of body parts. Approximately 35% (297/852) of the genes encoding “classical” ESP had a noticeable expression level in all body parts considered. At the same time, more than half (143/282) of genes encoding “non-classical” ESP presented this expression pattern. In both cases, the externa had the largest number of “specific” ESP: 325 and 56 “classical” and “non-classical” ESP, respectively. Dozens of ESP (101 “classical” and 35 “non-classical”) were common for the three interna parts considered.

Both “classical” and “non-classical” ESP were divided into families based on their sequence similarity. For “classical” ESP, 35 families contained two to four proteins, while only two “non-classical” ESP were combined into one family. Most (579/852) of the “classical” ESP matched the MetazSecKB database, the hits being, e.g., mannose-binding proteins, serine proteinase, and cuticle proteins (Table S5, *Extended data* ^71^). Only 58 “non-classical” ESP matched MetazSecKB, of which 35 were “uncharacterized proteins” (Table S5, *Extended data* ^71^). All ESP were also compared to the NeuroPep database, with which only 14 “classical” ESP had hits (Table S5, *Extended data* ^71^). The latter included cerebellin-1, kininogen-1, insulin-like growth factors I and II, neuroparsin-A, nucleobindin-2, and five representatives of the serpin family.

Figure 3c shows bioprocesses enriched with genes encoding “classical” ESP. Among the body-parts-specific bioprocesses were the “molting cycle” and “chitin-based cuticle development” in the externa, “positive regulation of cell communication” and “muscle organ development” in the growing interna part, “immune response” and “regulation of apoptotic signaling pathway” in the main trunk and the thoracic part of interna, respectively. The majority (21/34) of enriched bioprocesses were common for two or more body parts considered. For example, “regulated exocytosis” (externa and growing part), “mesoderm development” (the growing part and the main trunk), “cell recognition” (same), “proteolysis” (all body parts considered), “motor neuron axon guidance” (same), “cell adhesion” (same), and “neuron recognition” (externa and thoracic part of interna). The activity of the bioprocesses associated with the involvement of the nervous system is consistent with the fact that trophic rootlets of *P. reticulata* were enlaced by a network of host's neurons marked by a presence of a-tubulin and serotonin ( Figure 3d–f).

All ESP analysis results are presented in Table S5 ( *Extended data* ^71^).

### Significant differences between the TAI of body parts were revealed

**Figure 4.**
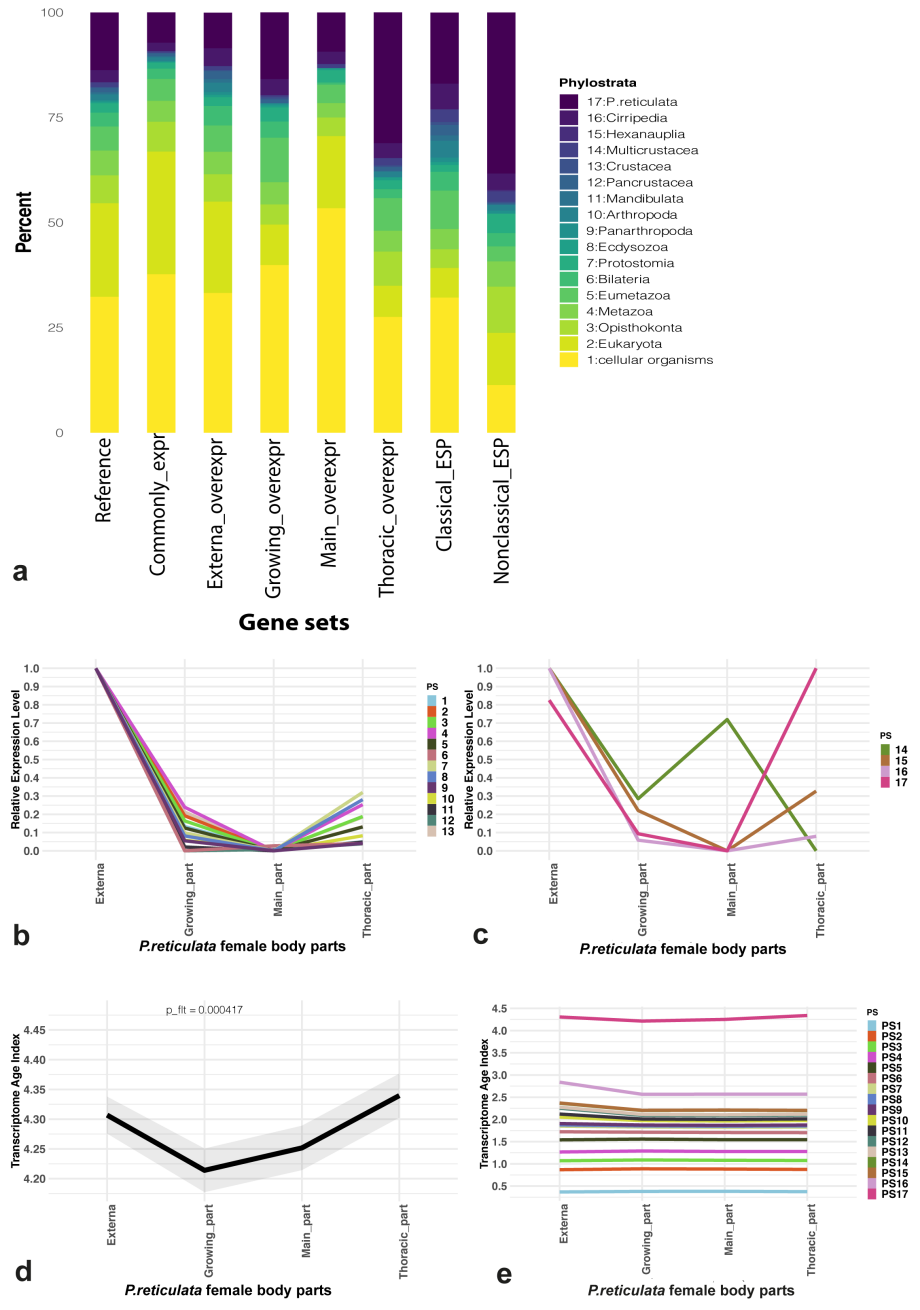
Phylostratigraphy and evolutionary transcriptomics results. ( **a**) Phylostratigraphic composition analysis results for *P. reticulata* reference gene set (“reference”), set of genes with noticeable (≥ 2 transcripts-per-million) expression in all female body parts considered (“common expr”), sets of genes over-expressed (externa/growing/main trunk/thoracic_overexpr) and sets of genes encoding potential “classical” (“classical_ESP”) and “non-classical” (“nonclassical_ESP”) excretory/secretory proteins (ESP). ( **b**, **c**) Relative mean expression levels of phylostrata which occurred “before” ( **b**) or “after” ( **c**) division of Crustacea. ( **d**) Transcriptome Age Indices (TAI) variation for female body parts considered. A lower TAI value describes an “older” transcriptome, whereas a higher TAI denotes a “younger” one. ( **e**) The cumulative phylostrata contribution to the final (global) TAI profile.

Almost all (12,618 / 12,620) *P. reticulata* genes were distributed across 17 phylostrata, i.e. sets of genes that coalesce to founder genes having a common phylogenetic origin ^72^ (Table S6, *Extended data*) The three largest phylostrata were “Cellular organisms” (32.34%, 4,082 genes), “Eukaryota” (22.25%, 2,809 genes), and species-specific ones (13.7%, 1,729 genes) ( Figure 4a). Less than 100 genes were assigned to “Ecdysozoa” (50 genes), “Panarthropoda” ^44^, “Mandibulata” (63 genes), “Crustacea” ^28^, and “Hexanauplia” ^6^. Phylostratum “Cirripedia”, which included two barnacles, *A. amphitrite* and *P. reticulata*, consisted of 363 genes.

The phylostratigraphy results were also used for composition analysis of various gene sets ( Figure 4a). More than half of the genes with expression ≥ 2 TPM in all body parts considered belonged to “Cellular organisms” and “Eukaryota” phylostrata. The proportion of species-specific genes in this set was approximately 7% (490/6,829). Noticeable differences in the contributions of different phylostrata to the sets of over-expressed genes were found. For example, approximately 31% (88/283) of such genes in the thoracic interna part belonged to the species-specific phylostratum. In contrast, in other parts of the body, the proportion of species-specific genes from the total number of over-expressed genes did not exceed 16%. A complex phylostratigraphic composition was also revealed for genes encoding both "classical" and "non-classical" ESP. “Cellular organisms” phylostratum made the greatest contribution to the “classical” ESP (32.16%), while the species-specific phylostratum contributed most to the “non-classical” ESP (38.3%).

We divided the phylostrata into two groups: before the divergence of Crustacea (from “Cellular organisms” to “Crustacea”) and after this event (from “Multicrustacea” to “ *P. reticulata*”). The relative expression patterns of the phylostrata are shown in Figure 4b, c. All phylostrata except the species-specific one had the highest relative expression in the externa, while the highest expression of the species-specific phylostratum was recorded in the thoracic part of the interna. At the same time, 14 out of 17 phylostrata had the least expression in the main trunk of the interna, the remaining three being “Bilateria”, “Pancrustacea”, and “Multicrustacea”.

One metric to quantify transcriptome conservation on a global scale is the TAI ^73^, which denotes the average transcriptome age throughout the biological process of interest ^64^. The TAI was measured for each part of the female *P. reticulata* body. The higher the value of the TAI, the greater the contribution of the “young” phylostrata. Significant differences between the TAI of different body parts were revealed. The TAI of the thoracic part of the interna was the highest (4.33), whereas the TAI of the growing part of the interna was the lowest (4.21) ( Figure 4d). The partial contribution of the “ *P. reticulata*” phylostratum to the TAI of the body part was about approximately three times greater than the contribution of any other phylostratum ( Figure 4e).

**Figure 5.**
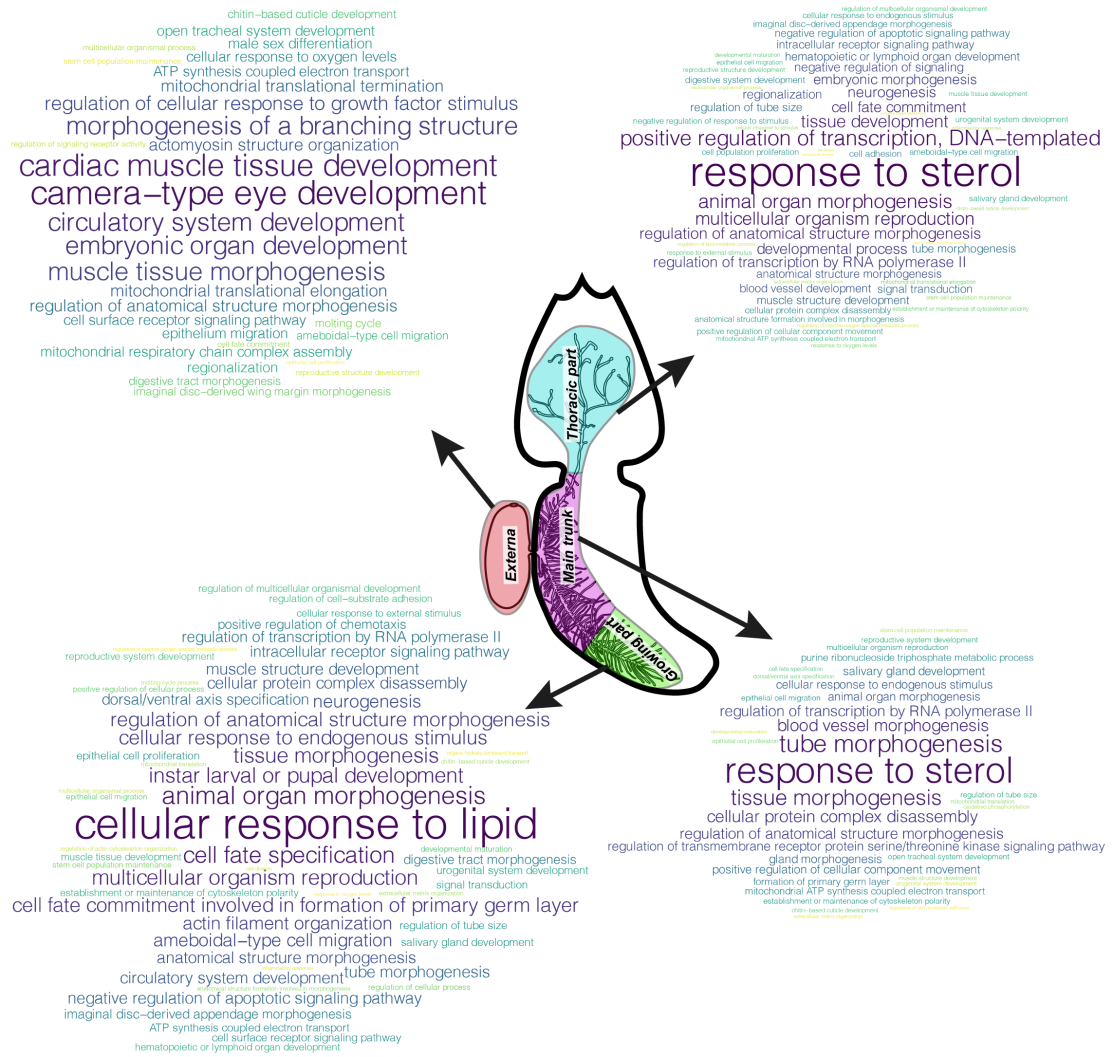
Gene set enrichment analysis (GSEA) results for top-500 annotated genes with the largest contribution to Transcriptome Age Index (TAI). Clouds of parental bioprocesses enriched with top-500 genes with both Gene Ontology (GO) annotation and large contribution to TAI of the body part under consideration. The more often the bioprocess was found in the list of enriched bioprocesses, the larger the word size.

We extracted the top-500 with the largest contribution to the TAI of body parts from the genes with the GO annotation and performed GSEA for these gene sets ( Figure 5). Among the “specific” bioprocesses were “embryonic organ development” and “male sex differentiation” in the externa, “cell fate commitment involved in the formation of primary germ layer” and “positive regulation of chemotaxis” in the growing part of the interna, “formation of primary germ layer” and “gland morphogenesis” in the main trunk of the interna, and “response to external stimulus” and “cell population proliferation” in its thoracic part. Only four bioprocesses were common for all the female body parts considered: “stem cell population maintenance”, “regulation of anatomical structure morphogenesis”, “chitin-based cuticle development”, and “animal organ morphogenesis”. Developmental processes were registered in each of the female body parts studied ( Figure 5).

Phylostratigraphy and evolutionary transcriptomics results are presented in Tables S6 and S7 ( *Extended data* ^74, 75^), respectively.

## Discussion

In this study, we obtained the first transcriptomes of a rhizocephalan and made a comparative analysis of different body parts of an adult female rhizocephalan. Our results are a step towards understanding the functioning of these highly specialized parasites and the trends of their evolutionary history. Below we discuss 1) how the molecular signatures helped us to verify the functional role of each part of the parasite’s body; 2) potential excretory/secretory proteins and their putative role in host-parasite interactions; 3) the trends of rhizocephalan evolution derived from the phylostratigraphy and evolutionary transcriptomics results.

We consider contamination identification and elimination in molecular biological studies of non-model parasitic organisms as one of the main challenging tasks. In our case, this challenge was aggravated by the fact that both the parasite and its host were crustaceans. This means that approaches based on database comparison could be less effective than usual at separating the reads from different sources. For this reason, we chose an MCSC algorithm, which classifies sequences based on the analysis of their properties. Based on the results of a preliminary orthogroup reconstruction (data not shown) and the reconstruction after decontamination and considering that there was an approximate 14% reduction in the number of duplicates of single-copy metazoan orthologues, we can assume that at least part of the signal from the host was removed. In further analyses, we focused on 12,620 protein-coding genes with a noticeable expression level. The results indicate that the reference gene set obtained in our study corresponds to those of other crustacean species in quality and completeness. The results of the analysis of orthogroups revealed that the proteome of *P. reticulata* was more similar to that of *Amphibalanus amphitrite* than to any other crustacean species involved in our study. *Amphibalanus amphitrite* belongs to Thoracica, a sister group of Rhizocephala. Thoracica barnacles are mostly free-living but also highly transformed crustaceans. A comparative analysis involving numerous representatives of these two sister taxa may uncover evolutionarily conservative mechanisms of transformation of adult rhizocephalans.

The body of a female rhizocephalan is divided into the externa and an extensive interna, which has different ultrastructural organization in different parts ( 76 and Miroliubov unpublished data). Our aim was to make a detailed record of the genome’s activity in different parts of the female body. Therefore, we used the soft threshold of 2 TPM as a condition for identifying genes whose expression contributed to the molecular signature of the sample under consideration. The results of the study of gene expression in different parts of the female body showed that: 1) slightly more than half of the identified protein-coding genes worked in all the examined parts of the body, 2) the lists of genes with an increased expression differed greatly between body parts, 3) the externa always clustered separately from the interna, although the differences were also found between the sites of the interna. These results suggest that the morphological heterogeneity of the female body is reflected in the spatial differences of gene expression (molecular heterogeneity).

However, we suggest the molecular signature of the body part may be considered as a derivative of the transcriptomes of all cell types included in the body region analyzed. It also depends on the organism’s response to various stimuli and conditions (for example, the host's immune response, O _2_ level and concentrations of different metabolites in the host’s hemolymph) ^15, 77^. Therefore, the more similar the cellular composition of body parts or the set of factors affecting them are, the more similar their molecular signatures will be. In our study all parts of the interna clustered together and were separate from the externa cluster. At the same time, the differences of the biological replicates of the externae could be explained by the fact that the externa contains embryos at different stages of development. The signal from the embryos was probably so strong that even the pooling of samples did not smooth out the differences.

Our results indicate that various processes are at work in different body parts of female rhizocephalan. Active developmental processes were registered in the externa, which could be expected considering that it contains numerous embryos, while active metabolism processes were recorded in the interna ( Figure 2c). These results are consistent with the classical concepts of the functional role of individual parts of the rhizocephalan body ^1, 2^.

However, some of our findings are at odds with the classical views. Previous morphological studies have postulated that the ovary is located in the visceral mass of the externa ^2^. However, the GSEA revealed that the female germ cells formed in the interna. Moreover, in histological sections we observed some cells floating in the central lumen of the main trunk, that looked like primary germ cells. Such cells have been described before ^78, 79^ but the authors assumed them to be stem cells, responsible for the formation of new buds of externae. Based on histological data and the GSEA results, we suggest that these cells are more likely to be female germ cells. We suppose that female germ cells begin and/or continue to form in the interna and then migrate to the externa, where they mature and are fertilized. In our opinion, the ovary of rhizocephalans is diffused within the interna, while the externa serves merely as a brooding chamber. 

Our finding of a “diffused” ovary prompts a reconsideration of the phenomenon of rhizocephalan “coloniality”. As noted above, some rhizocephalans form numerous externae. It has been suggested that each externa is a separate reproductive module, and the entire animal has been considered as a colony ^2, 78^. However, if the ovary is in fact diffused and scattered across the interna and if numerous externae are merely brooding chambers, the term “coloniality” does not seem suitable for Rhizocephala. This issue calls for further research with the use of molecular and morphological methods.

Regardless of whether a rhizocephalan barnacle is a colony or an individual organism, it has to communicate with the host via special excretory molecules involved in particular bioprocesses ^3^. We found that the composition of the potential excretome/secretome varied in different parts of the female rhizocephalan body and showed the character of the distribution of the secreted substances. For instance, in the externa there seems to be an active excretion/secretion of proteins involved in the storage of nutrients in the developing embryos as well as those involved in the molting cycle. At the same time, proteins responsible for muscle development were also among of the potential excretome/secretome of the growing part of the interna. These data confirm that the muscular system is formed in a growing tip of the main trunk ^76^.

Potential excretory/secretory proteins involved in neuron axon guidance were found in both the externa and the interna. While neuron axon guidance in the externa is probably associated with the offspring development, the evidence of this process in the interna is less expected and more interesting. A direct contact between the parasite and the host’s nervous system was shown in this study and in our previous research ^3^. We can expect the parasite to emit attracting signals, directing the host’s nervous system towards and along the interna. In addition, the excretory proteins involved in cell adhesion could also play an important role in the formation of a neural network around the rootlets. However, it cannot be ruled out that this transcriptome signal comes from the host tissues surrounding the rootlets of the parasite, since it is technically impossible to completely separate interna from the host’s tissues. Nevertheless, an intimate host-parasite interaction is an intriguing phenomenon requiring in-depth research.

The evolution of parasitic barnacles and their interactions with the hosts remains enigmatic ^80^. In particular, this concerns the molecular basis of both the formation of phenotypes at different stages of the rhizocephalan life cycle and the interaction of the parasite with the host. At the same time, the principle of genomic phylostratigraphy implies that the genome of every extant species retains parts of the picture of the evolutionary epochs ^72^. In order to determine how the *P. reticulata* gene set changed during evolution, we applied modern implementations of the phylostratigraphy to identify of gene groups with a common phylogenetic origin, called “phylostratum” or “phylostrata” in the plural form. The results of the phylostratigraphy analysis indicate that almost all *P. reticulata* genes were successfully distributed into 17 phylostrata. The third largest phylostratum was a species-specific one, which also probably includes genus- and family-specific orthologs. Given the transcriptome obtained in our study is the first reported rhizocephalan transcriptome, it is difficult to determine what percentage of proteins from this phylostratum belongs to each of these categories. Nevertheless, we are confident that a more detailed analysis of this particular phylostratum will allow us to identify the molecular basis of the Rhizocephala-specific biological traits. However, it should be kept in mind that genes need a cellular environment, the combined action of multiple other genes, as well as certain conditions to have an observable effect on organisms ^81^.

It should be noted that we analysed the reference gene set reconstructed based on the transcriptomic data, and the transcripts of poorly expressed or completely silent genes may be absent from our data. However, since we obtained transcriptomes of different regions of the female body and used a soft expression level threshold, we may be fairly sure that many genes active in the adult female were represented in the reference gene set.

Our results indicate that evolutionarily younger genes make a relatively large contribution to the signatures of the externa and the thoracic part of the interna. The transcriptional “youth” of the externa could be associated with the fact that the samples contained highly modified males ^2^. Our assumption is also based on the fact that we observed "male sex differentiation" among the lists of bioprocesses enriched by genes with the largest contributions to the TAI of externa. On the contrary, the signature of the thoracic part probably does not have any additional components. That is, we can assume that a high TAI value for this region may be associated with the evolutionary transformation of the region itself. We have already previously identified morphological heterogeneity of the entire body of an adult rhizocephalan female ^9, 76^. Given the differences between body regions in morphology, we also expected to find molecular and functional heterogeneity in the body of an adult female *P. reticulata*. The results obtained from the studies indicate that such heterogeneity manifests itself not only in the expression of individual genes and the activity of bioprocesses, but also in the contributions of various phylostrata to the molecular signatures of interna regions studied. Further research should be directed towards a more detailed study of the identified differences in TAI between interna regions. At the same time, we assume that, for example, the revealed transcriptional “oldness” of the growing interna region is presumably due to the activity of conservative processes, including those associated with the cell cycle.

The GSEA results for genes with the greatest contribution to TAI, both in the interna and in the externa, revealed many developmental processes. One may get the impression that rhizocephalans have an incomplete and endless metamorphosis. Taking into account that the adult female body originates from a fraction of the larval body, we are inclined to agree with the hypothesis suggested by Glenner and Høeg ^80^. It postulates that ancestors of rhizocephalans were filter-feeding epibiotic barnacles and the interna of an adult female originates from the part of the larval body homologous to the peduncle of a Goose barnacle, whose metamorphosis went the “wrong” way. The peduncle separated from the rest of the body and gave rise to the interna ^80, 82^. Nevertheless, a contemporary species can only serve as a proxy for an ancestral model. More transcriptomic/genomic data on other rhizocephalans are necessary for a reliable reconstruction of the evolutionary history of this unique group of parasites.

To sum up, the first comparative transcriptomic results for rhizocephalans brought us closer to understanding molecular mechanisms underlying the biology of these amazing parasites. We established the molecular and functional heterogeneity of the female rizocephalan body in addition to the known morphological one. A similarity was found between the set of protein-coding genes of the *P. reticulata* and that of a free-living representative of the sister taxon. Both bioinformatic data analysis and histological results indicated the presence of germ cells in the lumen of the interna, which casts doubt on the rhizocephalan coloniality. The molecular basis of the interaction between the nervous system of the host and the parasite's interna was determined. We established the differences between body parts in terms of phylostrata expression and their contribution to molecular signatures. Our results could indicate that rhizocephalans “got stuck in the metamorphosis” even in their reproductive stage. Our study can serve as a basis for further research on rhizocephalan evolution.

## Data availability

### Underlying data

 NCBI BioProject: *Peltogaster reticulata* Raw sequence reads, accession number: PRJNA798055.

Figshare: Table S1. Summary of paired-end read libraries preparation results, https://doi.org/10.6084/m9.figshare.19307486 ^66^

Figshare: Table S2. *Peltogaster reticulata* reference sequence set annotation results, https://doi.org/10.6084/m9.figshare.19307516 ^67^

Figshare: Table S3. Gene expression quantification and analysis results, https://doi.org/10.6084/m9.figshare.19307549 ^69^

### Extended data

Figshare: Table S4. Gene Set Enrichment Analysis (GSEA) results for the molecular signatures and sets of over-expressed genes, https://doi.org/10.6084/m9.figshare.19307570 ^70^

Figshare: Table S5. Potential excretory/secretory proteins analysis results, https://doi.org/10.6084/m9.figshare.19307594 ^71^

Figshare: Table S6. Phylostratigraphic affiliation analysis results for different set of sequences, https://doi.org/10.6084/m9.figshare.19307606 ^74^

Figshare: Table S7. Evolutionary transcriptomics results, https://doi.org/10.6084/m9.figshare.19307621 ^75^

Figshare: Figure S1. Phylogenetic relationships between the crustacean species based on orthologs analysis results, https://doi.org/10.6084/m9.figshare.19307444 ^68^
