# Supplementary material for: From head to rootlet: comparative transcriptomic analysis of a rhizocephalan barnacle Peltogaster reticulata (Crustacea: Rhizocephala)
Source: F1000Res. 2023 Jan 9;11:583. Originally published 2022 May 27. [Version 2] doi: 10.12688/f1000research.110492.2 (PMC9664023; doi:10.12688/f1000research.110492.2)
Supplement: Peer review history

# Peer review: Reviewer response for version 1

**DOI:** 10.5256/f1000research.122108.r153684

**Published:** 2022-11-14

**Authors:** Viatcheslav Ivanenko

**Affiliations:** 1 Shenzhen MSU-BIT University, Shenzhen, China; 2 Department of Invertebrate Zoology, Lomonosov Moscow State University, Moscow, Russian Federation; 3 Shirshov Institute of Oceanology, Russian Academy of Sciences, Moscow, Russian Federation

This manuscript is a completed work with new and significant scientific results. In my opinion, the manuscript is well written and illustrated, and the interpretation of the results raises no questions or doubts. The main remark concerns the fact that despite the uniqueness of many features of the biology and morphology of rhizocephalans, they are not the only highly modified crustaceans that parasitize crustaceans. In the first sentences of the manuscript or discussion, it would be appropriate to mention at least some poorly studied and highly modified copepods with root-like mouthparts that also parasitize other crustaceans. These copepods are belonging to the family Nicothoidae Dana, 1852-1853 and the synonymous family Choniostomatidae Hansen, 1886 (see Boxshall and Harrison, 1988 ^1^).

 Some phrases may not be very well written, but I am not a native English speaker to make edits.

Is the work clearly and accurately presented and does it cite the current literature?

Partly

If applicable, is the statistical analysis and its interpretation appropriate?

I cannot comment. A qualified statistician is required.

Are all the source data underlying the results available to ensure full reproducibility?

Yes

Is the study design appropriate and is the work technically sound?

Yes

Are the conclusions drawn adequately supported by the results?

Yes

Are sufficient details of methods and analysis provided to allow replication by others?

Yes

Reviewer Expertise:

Morphology, development, phylogeny and ecology of symbiotic crustaceans.

I confirm that I have read this submission and believe that I have an appropriate level of expertise to confirm that it is of an acceptable scientific standard.

## References

[1] : New nicothoid coepods (Copepoda: Siphonostomatoida) from an amphipod and from deep-sea isopods. *Bulletin of the British Museum (Natural History), Zoology* .1988;54(6) :285-299 Reference source

# Response

**Published:** 2022-12-27

**Authors:** Maksim Nesterenko

**Affiliations:** St. Petersburg University, Russian Federation

**We thank the reviewer for their comments, which are listed below (in italics) with our responses (in bold):**

*The main remark concerns the fact that despite the uniqueness of many features of the biology and morphology of rhizocephalans, they are not the only highly modified crustaceans that parasitize crustaceans. In the first sentences of the manuscript or discussion, it would be appropriate to mention at least some poorly studied and highly modified copepods with root-like mouthparts that also parasitize other crustaceans. These copepods are belonging to the family Nicothoidae Dana, 1852-1853 and the synonymous family Choniostomatidae Hansen, 1886 (see Boxshall and Harrison, 19881).*

**You are absolutely correct in pointing out that rhizocephalans are not the only ones showing amazing body transformation among Crustacea. We have added information about parasitic copepods at the end of the second paragraph of the discussion.**

*Some phrases may not be very well written, but I am not a native English speaker to make edits.*

**Even though for both authors of the article English is not their native language, we have tried to do our best to convey our thoughts as correctly and clearly as possible. However, we have tried to improve some of the wording.**

# Peer review: Reviewer response for version 1

**DOI:** 10.5256/f1000research.122108.r139230

**Published:** 2022-06-27

**Authors:** Andreas Hejnol, Ferenc Kagan

**Affiliations:** 1 Sars International Centre for Marine Molecular Biology, University of Bergen, Thormøhlensgate 55, 5006, Bergen, Norway, University of Bergen, Norway; 2 University of Jena, Jena, Germany; 3 University of Bergen, Bergen, Norway

This study provides the first glimpse into the transcriptomics of a parasitic rhizocephalan crustacean. The paper reads well, its coherent structure is easy to follow. The language used is appropriate for a publication and words are mindfully used when interpreting the results.

 The authors set out to provide transcriptomic data of the parasitic barnacle *Peltogaster reticulata*, a timely goal as this highly specialized group of crustaceans is heavily understudied. This, with the addition of the current study, appears to change as a novel chromosome level genome assembly has been recently made available for another rhizocephalan species ( *Sacculina carcini*). In this study different regions of the organism were selected, sequenced and their transcriptomic data were contrasted to each other. I found this approach sound to decipher the different roles of specialized body regions. I missed the description of the criteria used for separating the different body regions, especially for the internal structures.

 The authors successfully recognized and removed the challenge of biological contamination of the data. A thorough and careful preprocessing of the data was performed. The generation of a high-quality transcriptome assembly is pivotal for any project with similar aims. Therefore, the authors spend a tremendous amount of time assuring the quality of the assembly and annotation. I liked the attention to the details during these steps. With several filtering criteria, they arrive at a set of contigs which are plausible. From the quality assessment, I missed the report of the full BUSCO scores (if BUSCO scores are the reported scores), not just the single-copy ortholog and duplicate, but also fragmented and missing proportions. Furthermore, with such strong filtering steps, valid data is also discarded in parallel with noise. To alleviate this problem generally multiple assemblers with multiple k-mer sizes are utilized and redundancy across the assemblies is dealt with downstream of the assembly (eg. DOI: 10.7717/peerj.5428 or https://doi.org/10.111/1755-0998.13593).[ref-1] ^,^[ref-2] This approach in my experience will usually greatly improve the assemblies, an improvement which might marginally benefit the authors of this study.

 Next, the focus was shifted to quantifying the expression of genes in the body regions and finding differentially expressed genes among them. Gene ontological enrichment was performed on different sets of genes (molecular signature set, overexpressed set and different phylostratographic sets). The output of these analyses with the addition of histological data prompted the authors to hypothesize that female germ cells are formed in the internal trunk region and migrate from here to the externa part. Indeed, such results combined with previous reports hint towards this possibility, something which could be tested with an *in situ *hybridization for germ cell marker genes such as for example *vasa* or *nanos *(both of which are present in the assembled transcriptome).

 Using various *in silico *methods the authors outlined a set of potential excretory/secretory proteins. Several uncharacterized proteins are retrieved from this identification method, some of which could potentially be involved at the interface between parasite and host, as suggested by the authors. Unfortunately, without further validation, this suggestion persists as a testable hypothesis.

 Results from the phylostratographic analyses emphasize further the unique biology of parasitic barnacles. The interpretation of the results by the authors was legitimate. My concern with such approaches is their sensitivity to the BLAST algorithm (see a thorough description of the issue here: https://drostlab.github.io/myTAI/articles/Phylostratigraphy.html)

 Furthermore, I found the wording used for describing the datasets utilized in similarity searches confusing. How many species were used for the phylostratographic mapping and how many of them were metazoan?

 A rich resource of results was provided by the authors. With the descriptions provided the additional data is easily interpretable. At the moment the analyses conducted in this study are irreproducible without the custom scripts used by the authors. These should be made available for the community who wishes to replicate the study or to further investigate these intriguing parasitic barnacles.

 Overall I found this publication exciting and serving as a springboard for future studies on parasitic barnacle biology or evolutionary biology of parasitic life histories. If the authors complement the manuscript with the missing parts outlined above (criteria used for separating the body regions, phylostratography wording, missing scripts) the article would be greatly enhanced.

Is the work clearly and accurately presented and does it cite the current literature?

Yes

If applicable, is the statistical analysis and its interpretation appropriate?

Partly

Are all the source data underlying the results available to ensure full reproducibility?

Yes

Is the study design appropriate and is the work technically sound?

Yes

Are the conclusions drawn adequately supported by the results?

Yes

Are sufficient details of methods and analysis provided to allow replication by others?

Partly

Reviewer Expertise:

comparative transcriptomics in development and evolution

We confirm that we have read this submission and believe that we have an appropriate level of expertise to confirm that it is of an acceptable scientific standard.

# Response

**Published:** 2022-12-27

**Authors:** Maksim Nesterenko

**Affiliations:** St. Petersburg University, Russian Federation

**We thank the reviewer for their very helpful, clear, and pertinent remarks. Each original comment is in italics and our responses are in bold.**

*The authors set out to provide transcriptomic data of the parasitic barnacle Peltogaster reticulata, a timely goal as this highly specialized group of crustaceans is heavily understudied. This, with the addition of the current study, appears to change as a novel chromosome level genome assembly has been recently made available for another rhizocephalan species (Sacculina carcini). In this study different regions of the organism were selected, sequenced and their transcriptomic data were contrasted to each other. I found this approach sound to decipher the different roles of specialized body regions. I missed the description of the criteria used for separating the different body regions, especially for the internal structures.*

**We have added a more defined description of the way of separating different body parts in the Methods section.**

*The authors successfully recognized and removed the challenge of biological contamination of the data. A thorough and careful preprocessing of the data was performed. The generation of a high-quality transcriptome assembly is pivotal for any project with similar aims. Therefore, the authors spend a tremendous amount of time assuring the quality of the assembly and annotation. I liked the attention to the details during these steps. With several filtering criteria, they arrive at a set of contigs which are plausible. From the quality assessment, I missed the report of the full BUSCO scores (if BUSCO scores are the reported scores), not just the single-copy ortholog and duplicate, but also fragmented and missing proportions.*

**We refined the results of the comparison against the database of single-copy orthologs by indicating the percentages of orthologs that were duplicated, fragmented, or missing from our dataset. For example: “The comparison with a single-copy metazoan orthologues database revealed that in the reference protein set of *P. reticulata* 75.6% of the orthologues were assembled completely (Single: 70.4%; Duplicated: 5.2%), whereas 3.4% and 21% of the sequences were fragmented or absent, respectively.”**

*Furthermore, with such strong filtering steps, valid data is also discarded in parallel with noise. To alleviate this problem generally multiple assemblers with multiple k-mer sizes are utilized and redundancy across the assemblies is dealt with downstream of the assembly (eg. DOI: 10.7717/peerj.5428 or https://doi.org/10.111/1755-0998.13593).[ref-1],[ref-2] This approach in my experience will usually greatly improve the assemblies, an improvement which might marginally benefit the authors of this study.*

**We fully agree that the proposed method can indeed improve assembling results. Moreover, using multiple assemblers and re-running programs with different k-mer lengths was considered as a possible strategy in the early stages of planning a general analysis scheme based on the results presented in the TransRate publication (DOI: 10.1101/gr.196469.115). However, for the initial assembly, we opted for the Trinity program, which shows excellent assembly results even with default parameters. Now we are starting work on a new article, which will include new data, with the help of which we plan to improve the assembly presented in the current manuscript and refine the results obtained on its basis. As part of the new study, we also plan to test the approach you indicated in order to obtain the most complete and high-quality assembly of the transcriptome.**

*Next, the focus was shifted to quantifying the expression of genes in the body regions and finding differentially expressed genes among them. Gene ontological enrichment was performed on different sets of genes (molecular signature set, overexpressed set and different phylostratographic sets). The output of these analyses with the addition of histological data prompted the authors to hypothesize that female germ cells are formed in the internal trunk region and migrate from here to the externa part. Indeed, such results combined with previous reports hint towards this possibility, something which could be tested with an in situ hybridization for germ cell marker genes such as for example vasa or nanos (both of which are present in the assembled transcriptome).*

**Soon we are going to test our hypothesis by an *in situ *hybridization for germ cell marker genes. However, we plan to publish the results obtained in a separate article.**

*Using various in silico methods the authors outlined a set of potential excretory/secretory proteins. Several uncharacterized proteins are retrieved from this identification method, some of which could potentially be involved at the interface between parasite and host, as suggested by the authors. Unfortunately, without further validation, this suggestion persists as a testable hypothesis.*

**We fully agree that the presented results are preliminary and are aimed primarily at narrowing the range of potential targets for a more detailed study. Now we are working on it with the use of a proteomic approach and hope to publish the results soon in our new publications.**

*Results from the phylostratographic analyses emphasize further the unique biology of parasitic barnacles. The interpretation of the results by the authors was legitimate. My concern with such approaches is their sensitivity to the BLAST algorithm (see a thorough description of the issue here: https://drostlab.github.io/myTAI/articles/Phylostratigraphy.html)*

**Before starting the phylostratigraphic analysis, we carefully reviewed various materials, including the ones you mentioned. So, for example, according to this publication (https://doi.org/10.1093/molbev/msw284) BLAST is an appropriate and sufficiently sensitive tool in phylostratigraphic analysis that does not appear to introduce significant biases into evolutionary pattern inferences. Nevertheless, we agree that more complex comparisons of different methods are required to determine how correctly they detect distant homologs. By the way, as one of the promising directions for improving the strategy for phylostrata identification the Leapfrog method (doi:10.1101/gr.216226.116) proposed by your scientific group and based on the inclusion of «bridge» species can be considered.**

*Furthermore, I found the wording used for describing the datasets utilized in similarity searches confusing. How many species were used for the phylostratographic mapping and how many of them were metazoan?*

**In total, in addition to the *P.*** ***reticulata,*** **139 species were included in the analysis, of which 44 were representatives of the Metazoa. We not only have provided this information in the Methods but have also included a phylogenetic tree of all species considered in the Supplementary materials. Thank you very much for pointing out to us that we missed this important information.**

*A rich resource of results was provided by the authors. With the descriptions provided the additional data is easily interpretable. At the moment the analyses conducted in this study are irreproducible without the custom scripts used by the authors. These should be made available for the community who wishes to replicate the study or to further investigate these intriguing parasitic barnacles.*

**All custom Python and R scripts used in the study are publicly available: https://github.com/maxnest/From_head_to_rootlet**
